# First-Principles Study of the Heterostructure, ZnSb Bilayer/h-BN Monolayer for Thermoelectric Applications

**DOI:** 10.3390/ma18020294

**Published:** 2025-01-10

**Authors:** Zakariae Darhi, Larbi El Farh, Ravindra Pandey

**Affiliations:** 1 Department of Physics, Mohamed 1st University, Oujda 60000, Morocco; zakariae.darhi@ump.ac.ma; 2 Department of Physics, Michigan Technological University, Houghton, MI 49931, USA; pandey@mtu.edu

**Keywords:** ZnSb bilayer, h-BN monolayer, vdW heterostructure, thermoelectric, DFT

## Abstract

ZnSb is widely recognized as a promising thermoelectric material in its bulk form, and a ZnSb bilayer was recently synthesized from the bulk. In this study, we designed a vertical van der Waals heterostructure consisting of a ZnSb bilayer and an h-BN monolayer to investigate its electronic, elastic, transport, and thermoelectric properties. Based on density functional theory, the results show that the formation of this heterostructure significantly enhances electron mobility and reduces the bandgap compared to the ZnSb bilayer, thereby increasing its power factor. These findings highlight the potential of the h-BN monolayer–supported ZnSb bilayer heterostructure in thermoelectric applications, where maximizing energy conversion efficiency is essential.

## 1. Introduction

The unique planar structure and physical properties of the vertical van der Waals (vdW) heterostructure have made them a key focus of research, driven by their potential in novel applications, including gas sensor [1], catalysis [2], energy storage [3], and thermoelectric applications [4]. Among these materials, hexagonal Boron Nitride (h-BN) is one of the most studied two-dimensional (2D) materials, serving as the substrate in heterostructures like InSe/h-BN [5], GaS/h-BN [6], W$S_{2}$/h-BN [7], and Ti$O_{2}$/h-BN [8]. When combined with other 2D materials, h-BN enhances the overall properties of a heterostructure. For instance, it has been reported that W$S_{2}$-based heterostructures on the h-BN monolayer exhibit significantly higher charge carrier mobility compared to those based on other substrates [9].

ZnSb is widely recognized as a promising thermoelectric material in its bulk form, with extensive research focused on optimizing its thermoelectric performance [10,11,12,13]. A ZnSb bilayer was synthesized from the bulk using an alkali metal alloying process followed by ion etching techniques [14]. This achievement has brought significant interest among researchers to explore the physical properties of this material, aiming to uncover new functionalities [15,16,17]. Notably, 2D ZnSb exhibits intriguing characteristics, including its robust topological nature [18,19].

Here, we systematically investigate the transport and thermoelectric properties of the ZnSb bilayer for the first time and analyze the impact of combining an h-BN monolayer as a substrate on its electronic, elastic, transport, and thermoelectric properties. Our results demonstrate a significant enhancement in electron mobility and elastic stiffness in the heterostructure, reflecting its potential for various applications.

## 2. Materials and Methods

First-principles calculations were performed using density functional theory (DFT) implemented in the Vienna Ab initio Simulation Package (VASP), version 6.4.1 [20,21,22,23,24]. We employed the projector-augmented wave (PAW) method [25] and the Perdew–Burke–Ernzerhof (PBE) exchange-correlation functional [26], with a kinetic energy cut-off set at 500 eV. The van der Waals interactions were included using Grimme’s empirical dispersion correction method (D3) [27], chosen for its reliability in capturing the structural and electronic properties of similar systems [28]. The convergence criteria for the forces and energy were set to 0.5 × $10^{-3}$ eV/Å and $10^{-8}$ eV, respectively. The Monkhorst–Pack scheme was used to generate a grid of k-points for sampling the Brillouin zone [29]. The k-point grid was 7 × 11 × 1 for the ZnSb bilayer and 8 × 13 × 1 for the heterostructure. The vacuum region in the z-direction was 25 Å and 30 Å for the ZnSb bilayer and ZnSb/h-BN heterostructure, respectively. The electronic transport properties were calculated using the Constant Relaxation Time Approximation (CRTA) within the BoltzTraP code [30]. with a k-point grid of 25 × 44 × 1.

## 3. Results

### 3.1. ZnSb Bilayer

#### 3.1.1. Structure and Stability

**Figure 1:**
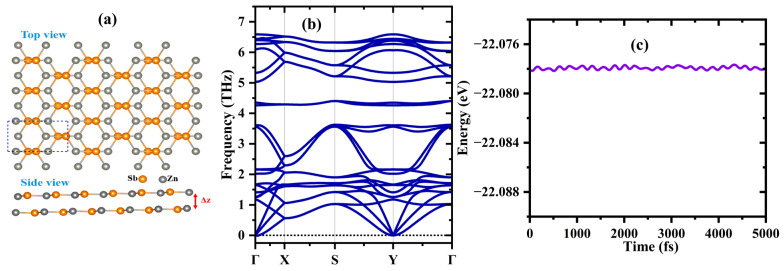
(**a**) A ball-and-stick model of the bilayer configuration in which the rectangular unit cell is indicated with the dashed blue line. Atomic color coordinates: Zn: orange, Sb: gray. (**b**) Phonon dispersion curve, and (**c**) total energy fluctuation as a function of the time step of the ZnSb bilayer.

The bilayer structure of ZnSb was formed by stacking relaxed ZnSb monolayers that belong to the hexagonal symmetry having a P3m1 space group, with the top layer shifted by a translation along the b-direction, similar to an AB-like stacking configuration. Note that this bilayer configuration was predicted to be energetically stable [19]. In Figure 1a, the dashed rectangle represents the unit cell of the bilayer, consisting of 4 Zn/Sb atoms. The calculated lattice parameters and the interlayer distance of the bilayer are a = 7.89 Å, b = 4.57 Å. The interlayer distance is 2.84 Å, and the near-neighbor distance ($d_{Zn-Sb}$) is 2.64 Å. The calculated binding energy is 2.19 eV with respect to the constituent monolayers. These values are consistent with previously reported theoretical results [18,19].

Next, we assess the thermal and dynamical stabilities of the bilayer configuration to confirm its stability further. The thermal stability was evaluated through ab initio molecular dynamics (AIMD) simulations at a finite temperature of 300 K. The temperature was controlled using the Andersen thermostat within the NVT ensemble, employing a time step of 1.0 fs for a total simulation time of 5 ps with a 3 × 5 × 1 k-point mesh. The simulation results are presented in Figure 1c, in which the energy fluctuations over time steps become small, leading to the conclusion that ZnSb is thermally stable. Additionally, we calculated the phonon dispersion spectra of a (2 × 2 × 1) supercell along the high-symmetry directions of the Brillouin zone using density functional perturbation theory (DFPT) [31] and the rotational invariance conditions to correct the forces using the HiPhive package (https://hiphive.materialsmodeling.org/, accessed on 7 January 2024) [32]. As shown in Figure 1b, the absence of imaginary phonon modes over the entire path emphasizes the dynamic stability of the ZnSb bilayer.

#### 3.1.2. Electronic Properties

**Figure 2:**
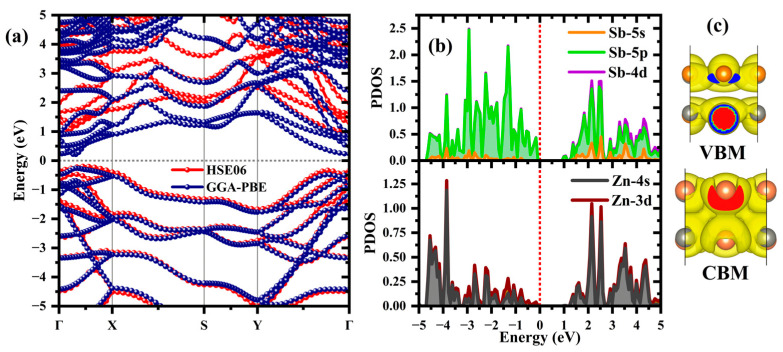
The (**a**) total band structure, (**b**) projected density of states using the HSE06 hybrid functional, and (**c**) side view of the band-decomposed charge densities corresponding to the VBM and CBM of the ZnSb bilayer, calculated using the HSE06 hybrid functional, with an isosurface value of 0.00195 ${e/Bohr}^{3}$.

The electronic structure of the ZnSb bilayer, represented in Figure 2a, reveals an indirect band gap of 0.55 eV using GGA-PBE. The valence band maximum (VBM) and conduction band minimum (CBM) are located between the X and Γ points within the Brillouin zone. Including spin–orbit coupling (SOC) (see Figure S1 in the Supplementary Materials), a shrink in the band gap is observed, reflecting the influence of SOC on the electronic properties (Figure S1). The calculated band gap aligns with previously GGA calculations: 0.55 eV without SOC and 0.45 eV with SOC [19]. To obtain a more accurate estimation of the band gap, hybrid functional calculations were employed [33], yielding a band gap energy of 1.19 eV. The partial charge densities corresponding to VBM (Figure 2b) suggested that Sb-p orbitals primarily contribute to the states near the Fermi level, with some contributions from the Zn-d orbitals. The calculated band-composed charge densities [34] show their localization within each layer for VBM. On the other hand, the CBM charge densities are delocalized throughout the bilayer, indicating that electrons are free to move and contribute to conductivity in the bilayer.

### 3.2. ZnSb Bilayer/h-BN Monolayer Heterostructure

#### 3.2.1. Structure and Stability

**Figure 3:**
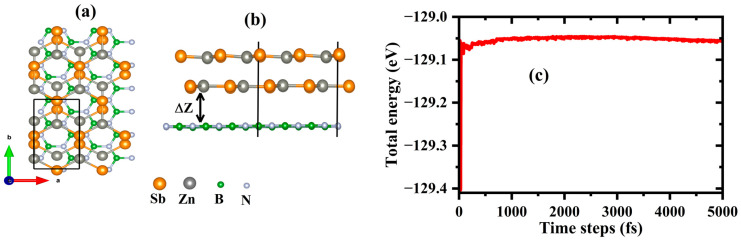
The schematic structure, viewed along the (**a**) c-direction (top view) and (**b**) a-direction (side view) of the unit cell of the heterostructure consisting of a ZnSb bilayer and h-BN monolayer. The interlayer distance (∆ z = 3.5 Å) is defined as the perpendicular (*z*-axis) distance between the reference atoms of adjacent layers from the optimized structure. (**c**) total energy vs. time relationship obtained from AIMD simulation. The black box represents the unit cell of the heterostructure.

Before designing the heterostructure, we fully optimized the individual structures of the ZnSb bilayer and the h-BN monolayer. Following the assembly of the heterostructure, a full structural relaxation was performed to ensure stability and minimize interlayer forces. The schematic structure, viewed along the c-direction (top view) and a-direction (side view) of the unit cell of the heterostructure, is shown in Figure 3a. To ensure the stability of the heterostructure, the interface strain is minimized by reducing the lattice mismatch between the constituent monolayers. This alignment was achieved by scaling the b-axis of the h-BN lattice, which has a P6-m2 space group, using a 1 × 3 × 1 supercell. This resulted in a lattice mismatch of 5.1% between the ZnSb bilayer and the h-BN monolayer.

The calculated lattice parameters are 4.406 Å for a and 7.62 Å for b with the interlayer distance, ∆ z, of 3.5 Å. The binding energy is calculated using the following equation:

(1) $$E_{binding}=\frac{E_{ZnSb/h-BN}-E_{ZnSb}-E_{h-BN}}{A_{0}},$$

Here, $E_{ZnSb/h-BN}$*,* $E_{ZnSb}$*,* and $E_{h-BN}$ represent the energy of the ZnSb/h-BN, ZnSb bilayer, and h-BN monolayer, respectively, and $A_{0}$ is the area of the interface. A negative value of 20.16 (meV/${Å}^{2}$), which falls within the range of typical van der Waals heterostructures (13–21 meV/${Å}^{2}$) [35], indicates the feasibility of experimentally synthesizing this heterostructure. Furthermore, the stability of the designed vdW heterostructure was evaluated using ab initio molecular dynamics (AIMD) simulations at 300 K. The simulation parameters, including a k-mesh of 7 × 4 × 1, were consistent with those used for the bilayer system. The behavior depicted in Figure 3b, where the oscillation of the energy around the equilibrium energy remains stable and very small (≈0.01 eV), confirms the dynamical stability of ZnSb/h-BN.

#### 3.2.2. Electronic Properties

**Figure 4:**
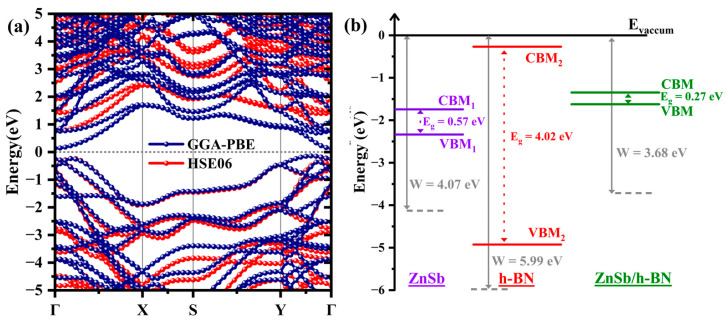
(**a**) The calculated band structure with PBE (navy) and HSE06 (red) calculations and (**b**) the band alignment for the ZnSb bilayer/h-BN monolayer heterostructure.

Figure 4a shows the band structure of the heterostructure in which the CBM is at Γ, and VBM is located between the Γ and X points, indicating an indirect bandgap of 0.27 eV (GGA-PBE) and 0.89 eV (HSE06). Compared to the band gaps of the constituents (i.e., 0.55 eV for the ZnSb bilayer and 4.55 eV for the h-BN monolayer using GGA-PBE), the bandgap of the heterostructure is significantly reduced primarily due to the alignment of the energy bands within the heterostructure, as shown in Figure 4b. The band alignment can be classified as a type II band alignment since the h-BN monolayer has a lower (higher) VBM (CBM) than the ZnSb bilayer; this means the electrons will accumulate on the h-BN monolayer, and holes will migrate to the ZnSb bilayer. This kind of interface is more suitable for solar cells.

**Figure 5:**
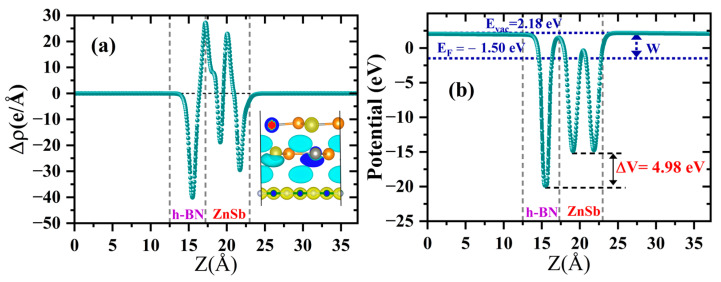
(**a**) The planar-averaged ∆ρ (z) and top view of ∆ρ(z) where the cyan and yellow areas indicate electron depletion and accumulation, respectively, with an isosurface value of 0.230 ${e/Bohr}^{3}$ and (**b**) the average electrostatic potential along the *z*-axis for the heterostructure.

To analyze the interlayer charge transfer during the formation of the heterostructure, the charge density difference, ∆ρ(z), along the z direction, with respect to its constituents, is plotted in Figure 5a, where the yellow color indicates the charge accumulation and the cyan indicates the depletion region. Also, analysis of Bader charges suggests a small charge transfer of about 0.03 e from the ZnSb bilayer to the h-BN monolayer. Note that the work function W is a critical tool to study the interfacial charge transfer in the heterostructure. The work function is defined using the following equation [36]:

(2) $$W=E_{vac}-E_{F},$$

where $E_{vac}$ is the electrostatic potential of the vacuum energy level, and $E_{F}$ represents the Fermi level. The work function of the heterostructure is 3.68 eV. On the other hand, the work functions of the h-BN monolayer and ZnSb bilayer are 4.07 and 5.99 eV, respectively, confirming that electrons flow from the ZnSb bilayer to the h-BN monolayer. This establishes a strong electric field at the interface, resulting in a significant drop in the electrostatic potential (ΔV = 4.98 eV) in the region of the h-BN monolayer (see Figure 5b).

### 3.3. Transport Properties

The carrier mobilities and relaxation time can be estimated using the deformation potential (DP) theory proposed by Bardeen and Shockley [37]. This theory has been extensively applied to predict the transport properties of 2D materials using the following formula:

(3) $$\mu_{2D}=\frac{{e\hbar }^{3}C_{2D}}{K_{B}Tm^{*}\sqrt{m_{d}}{\left(E_{l}\right)}^{2}},$$

In this context, ℏ is the reduced Planck constant, and $k_{B}$ is the Boltzmann constant. $m^{*}=\hbar^{2}{\left[\frac{\partial^{2}E(k)}{{\partial k}_{x}{\partial k}_{y}}\right]}^{-1}$ and $m_{d}=\sqrt{m_{x}^{*}m_{y}^{*}}$ are the effective mass along the transport direction and average effective mass, respectively.

Table 1 lists the computed m* values for electrons and holes along the x and y directions. $C_{2D}$ represents the elastic modulus derived from the equation $C_{2D}=\frac{1}{A_{0}}\frac{\partial^{2}E}{{\partial \varepsilon }^{2}}$, where $A_{0}=a_{0}b_{0}$ is the area of the unit cell. E is the total energy after stretching and compressing the lattice vectors using a strain ε ranging from −2% to 2%. The quadratic polynomial fit of energy versus strain is given in the Supplementary Materials (see Figures S2 and S3), and the elastic modulus values for electrons and holes in both directions are calculated and listed in Table 1.

The deformation potential $E_{l}$ is then calculated as $dE_{edge}/d\varepsilon$, equivalent to the slope of the fitting lines, where $E_{edge}$ is the conduction or valence band edge (see the linear fitting in Figures S1 and S2). Finally, by combining all the previous physical quantities ($E_{l}$, $C_{2D}$, and $m^{*}$), we estimated the relaxation time using the following equation:

(4) $$\tau_{2D}=\frac{\mu_{2D}m^{*}}{e},$$

The calculated electron mobility of the ZnSb bilayer is 217.8 ${cm}^{2}{\cdot V}^{-1}{\cdot s}^{-1}$ along the x-direction and 297.8 ${cm}^{2}{\cdot V}^{-1}{\cdot s}^{-1}$ along the y-direction. Meanwhile, the hole mobility is significantly higher, with values of 1004.5 ${cm}^{2}{\cdot V}^{-1}{\cdot s}^{-1}$ along the x-direction and 703 ${cm}^{2}{\cdot V}^{-1}{\cdot s}^{-1}$ along the y-direction, reflecting that the electrons prefer to migrate along the y-direction, while holes tend to migrate along the x-direction.

On the other hand, the electron mobility and relaxation time were significantly higher along the x-direction in the heterostructure, notably due to the enhanced elastic modulus and relatively lower deformation potential than those of the ZnSb bilayer. A larger effective mass and high deformation potential for holes lead to attenuation of the relaxation time and hole mobility in the ZnSb bilayer/h-BN monolayer heterostructure.

### 3.4. Thermoelectric Performance

**Figure 6:**
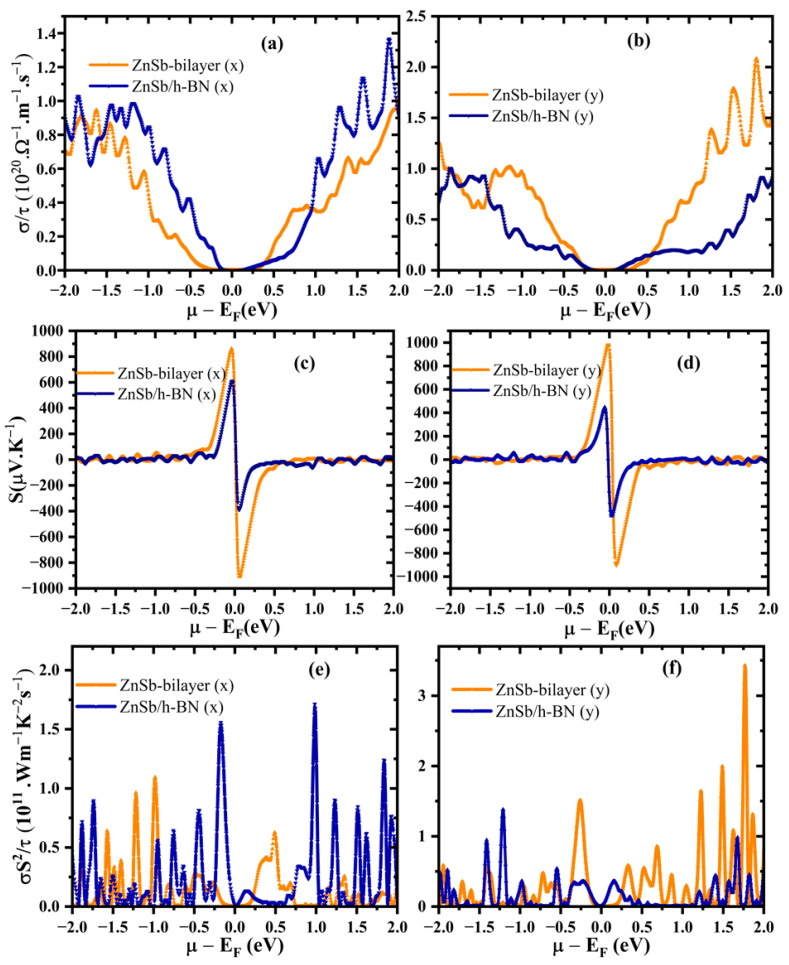
(**a**,**b**) Electrical conductivity, (**c**,**d**) Seebeck coefficient, (**e**,**f**) power factor with chemical potential at 300 K of the ZnSb bilayer and the heterostructure.

Achieving good thermoelectric performance requires a high-power factor, $PF=\sigma S^{2}$, in which *σ* represents electrical conductivity and S is the Seebeck coefficient. The Seebeck coefficient, electrical conductivity, and power factor are collectively presented in Figure 6 as a function of the chemical potential for bilayer ZnSb and its heterostructure with an h-BN monolayer. The chemical potential represents the doping level of the material, where negative values correspond to p-type doping and positive values indicate n-type doping.

The calculation of electrical conductivity per relaxation time shows a pronounced anisotropy for both the bilayer and the heterostructure, as shown in Figure 6a,b. In the heterostructure, the highest conductivity occurs along the x-direction under positive chemical potential, with a peak around 1.87 eV. In contrast, the bilayer exhibits the highest electrical conductivity in the n-type doping region along the y-direction. Despite the bilayer’s high electrical conductivity in the y-direction, the heterostructure demonstrates enhanced electrical conductivity along the x-direction. This suggests that the layered configuration may improve charge transport along the x-direction and give rise to a promising PF factor in the x-direction for the heterostructure.

The dependence of the Seebeck coefficient on chemical potential is shown in Figure 6c,d. The Seebeck coefficient in the heterostructure is lower than the bilayer in both directions. This reduction can be explained by the inverse relationship between the Seebeck coefficient, the number of layers, carrier concentration near the Fermi level, and the enhanced bipolar conduction effect caused by the narrower bandgap in the heterostructure. A large Seebeck coefficient around the Fermi level indicates that S can be enhanced through p- or n-type doping. The maximum |S| for the ZnSb bilayer (909 µV/K) is significantly higher than the reported experimental value of the bulk system (196 µV/K) [38]. Figure 6e,d show that the vdW heterostructure demonstrates an optimized power factor along the x-direction, particularly for the n-type doping. Our findings regarding the electronic properties (projected density of states) of the heterostructure show that the states near the Fermi level, primarily contributed to by the metallic atoms (Zn and Sb), enhance the electrical conductivity (refer to Figure S4). This improvement in conductivity is a key factor that can significantly boost the power factor of the device, making the heterostructure highly suitable for thermoelectric applications.

### 3.5. Mechanical Properties

Predicting the elastic properties may provide insights into understanding such enhancement in the transport properties of the heterostructure. The mechanical properties were calculated using the strain energy method, in which the material was under the strain, ranging from −2% to +2%. The elastic constants are obtained using equation [39]:

(5) $$C_{ij}=\frac{1}{A_{0}}\left(\frac{\partial^{2}∆E}{\partial \varepsilon_{i}\varepsilon_{j}}\right),$$

where $∆E=\frac{A_{0}}{2}\left(C_{11}\varepsilon_{1}^{2}+C_{22}\varepsilon_{2}^{2}+{2C}_{12}\varepsilon_{1}\varepsilon_{2}+C_{66}\varepsilon_{6}^{2}\right)$ is the second partial derivative of strain energy.

The elastic stiffness constants, Young’s modulus, and Poisson’s ratio of the ZnSb bilayer and the heterostructure are listed in Table S1. Based on the values of $C_{ij}$ and the Born–Huang stability criteria for rectangular 2D system ($C_{11},\;C_{66}>0$, $C_{11}C_{22}>C_{12}^{2}$) [40], both the bilayer and the heterostructure are predicted to be mechanically stable. The elastic constants and Young’s modulus of the heterostructure are higher than those of the bilayer, indicating an enhanced resistance to strain and greater tunability in flexibility. The Young’s modulus values are 316.2 N/m and 324.9 N/m along the x- and y-directions, respectively, which are comparable to previously reported values for Black Phosphorene (BP)/MoSSe (307.8 N/m), a strong candidate for thermoelectric applications [41].

## 4. Summary

We have investigated the properties of the ZnSb bilayer and the effects of the heterostructure on tuning the transport properties using DFT. The designed heterostructure of the ZnSb bilayer with the h-BN monolayer is predicted to be stable, exhibiting a Type-II semiconductor. Notably, the heterostructure shows higher mobility and a lower deformation potential, resulting in an improved power factor. These findings provide insights into the ZnSb bilayer/h-BN monolayer as a promising candidate for thermoelectric applications.

## Figures and Tables

**Table 1:** The calculated effective mass (m*), deformation potential ($E_{l}$), elastic modulus ($C_{2D}$), carrier mobility (μ), and the relaxation time (τ) of the bilayer ZnSb and the heterostructure.

Direction	Structure	Carrier Type	$m^{*}(m_{0})$	$E_{l}$ (eV)	$C_{2D}$ (N·m^−1^)	μ (cm^2^·V^−1^·s^−1^)	τ(fs)
Γ→X	ZnSb bilayer	electron	0.318	−1.52	62.8	217.8	27.9
		hole	0.225	−6.63	62.8	1004.7	181.7
	ZnSb/h-BN heterostructure	electron	1.425	−0.69	342.1	14,107.7	11,430.5
		hole	1.219	−3.44	342.1	397.6	275.6
Γ→Y	ZnSb bilayer	electron	0.353	−7.59	68.5	297.8	192.3
		hole	0.481	−4.24	68.5	703.0	59.7
	ZnSb/h-BN heterostructure	electron	0.402	−4.23	345.1	1350.0	308.6
		Hole	1.324	−9.18	345.1	51.8	39.3

## Data Availability

The original contributions presented in this study are included in the article/Supplementary Materials. Further inquiries can be directed to the corresponding author.

## Supplementary Materials

The following supporting information can be downloaded at: https://www.mdpi.com/article/10.3390/ma18020294/s1, Figure S1: Calculated band structure of ZnSb-bilayer using PBE+SOC; Figure S2: Calculated total energy as function of applied uniaxial strains for (a) ZnSb-bilayer and (b) ZnSb/h-BN heterostructure; Figure S3: Valence and conduction band-edges energy as function of applied uniaxial strains for (a) ZnSb-bilayer and (b) ZnSb/h-BN heterostructure; Figure S4: Partial Density of States (PDOS) of the heterostructure; Figure S5: Calculated figure of merit ZT_e_ at 300 K of the ZnSb bilayer and the heterostructure; Table S1: The calculated elastic constants ($C_{ij}$), Young’s modulus (Y), and Poisson ratio (ν) of the ZnSb bilayer and the ZnSb bilayer/h-BN monolayer heterostructure. Reference [42] are cited in the supplementary materials.
