# Supplementary material for: First-Principles Study of the Heterostructure, ZnSb Bilayer/h-BN Monolayer for Thermoelectric Applications
Source: Materials (Basel). 2025 Jan 10;18(2):294. doi: 10.3390/ma18020294 (PMC11767080; doi:10.3390/ma18020294)
Supplement: Supplementary file 1 [file materials-18-00294-s001.zip › materials-3332137 - supplementary.pdf]

Supplementary Material

First-principles study of the heterostructure, ZnSb bilayer/h-BN monolayer for Thermoelectric Applications

Zakariae Darhi[1], Larbi El Farh[1], Ravindra Pandey[2]

[1]*Mohamed 1st University, Department of Physics, Oujda, 60000, Morocco*

[2]*Michigan Technological University, Department of Physics, Houghton, MI, 49931, USA*

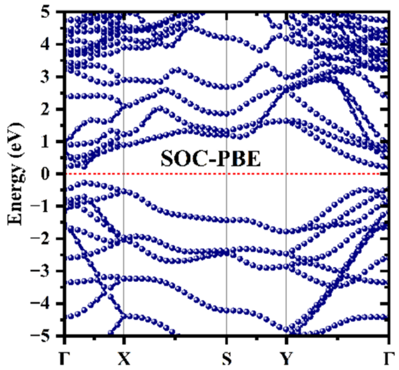

Figure S1 Calculated band structure of ZnSb-bilayer using PBE+SOC.

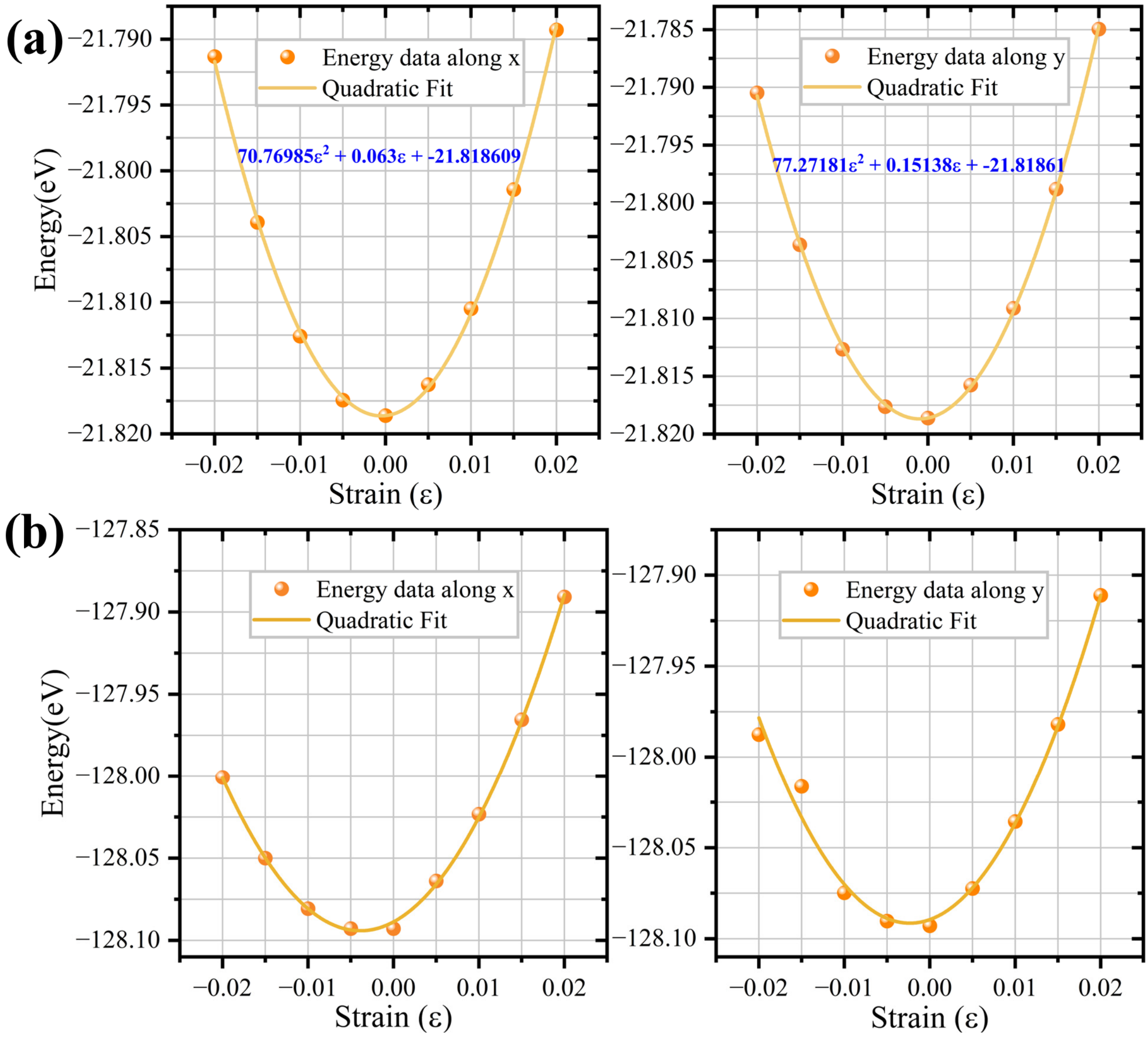

Figure S2 Calculated total energy as function of applied uniaxial strains for (a) ZnSb-bilayer and (b) ZnSb/h-BN heterostructure.

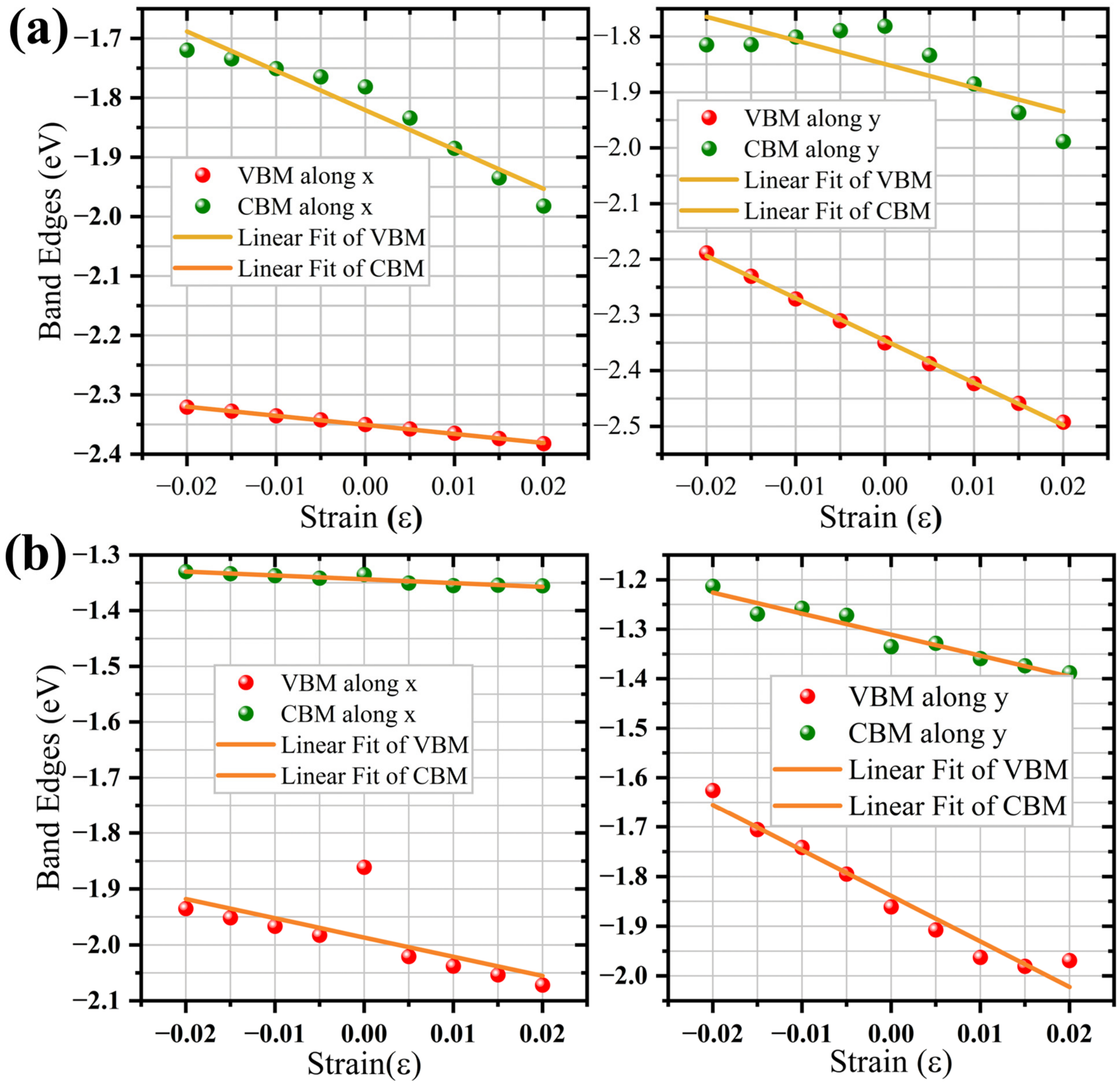

Figure S3 Valence and conduction band-edges energy as function of applied uniaxial strains for (a) ZnSb-bilayer and (b) ZnSb/h-BN heterostructure.

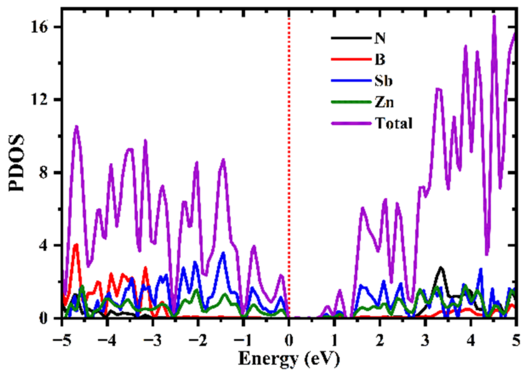

Figure S4. Partial Density of States (PDOS) of the heterostructure.

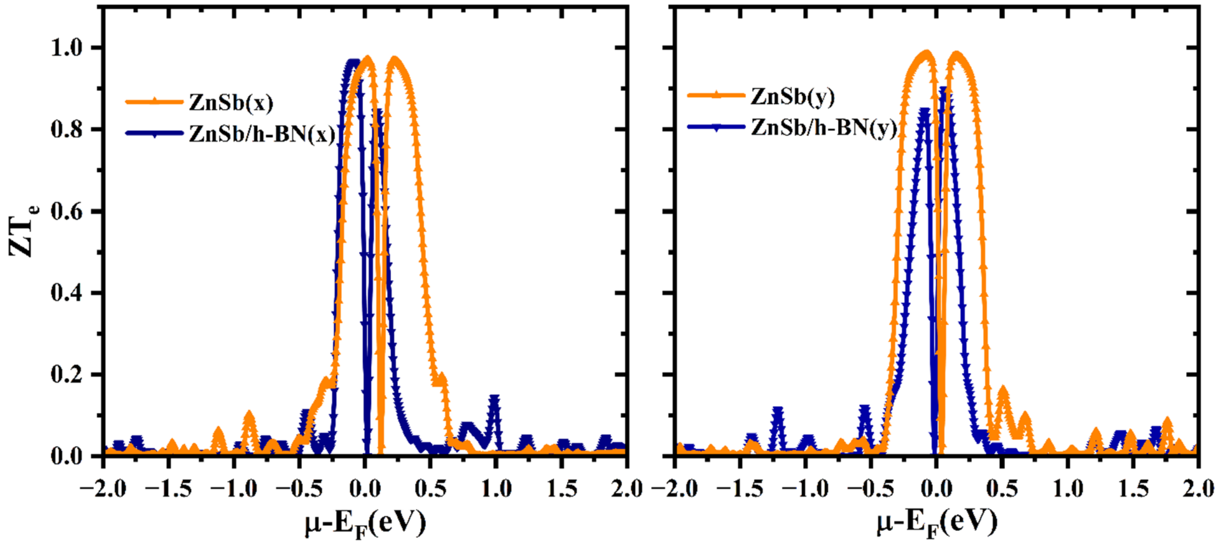

Figure S5. Calculated figure of merit ZT_e at 300 K of the ZnSb bilayer and the heterostructure.

Table S1. The calculated elastic constants (C_{ij}), Young's modulus (Y), and Poisson ratio (v) of the ZnSb bilayer and the ZnSb bilayer/h-BN monolayer heterostructure.

	ZnSb bilayer	h-BN monolayer [1]	ZnSb/h-BN Heterostructure
$\mathbf{C_{11}}$(N/m)	63.69	289.4	346.31
$\mathbf{C_{12}}$(N/m)	24.8	65.2	85.01
$\mathbf{C_{22}}$(N/m)	98.38	289.4	337.03
$\mathbf{C_{66}}$(N/m)	27.57	112.1	129.21
$\mathbf{Y_x}$(N/m)	57.44		316.16
$\mathbf{Y_y}$(N/m)	88.73		324.87
$\mathbf{\nu_{xy}}$(N/m)	0.25		0.24
$\mathbf{\nu_{yx}}$(N/m)	0.39		0.25

1. Pu, C.-Y.; Lv, L.-X.; Zhou, D.-W.; Yu, J.-H.; Tang, X. Structure, Electronic, and Mechanical Properties of Three Fully Hydrogenation h-BN: Theoretical Investigations. *Communications in Theoretical Physics* **2019**, *71*, 1363.
